# Olive mill wastewater treatment using coagulation/flocculation and filtration processes

**DOI:** 10.1016/j.heliyon.2024.e40348

**Published:** 2024-11-13

**Authors:** Layla Moustafa Fleyfel, Joseph Matta, Nicole Fakhoury Sayegh, Nasma Hamdi El Najjar

**Affiliations:** a Saint Joseph University of Beirut, Faculty of Pharmacy, Department of Nutrition, Medical Sciences Campus, Damascus Road, P.O.B. 11-5076, Riad Solh Beirut, 1107 2180, Lebanon; b Industrial Research Institute (IRI), Lebanese University Campus, Hadeth Baadba, Lebanon

**Keywords:** Olive mill wastewater, Coagulation/flocculation, Filtration, Phenolic compounds

## Abstract

Olive mill wastewater (OMWW), a pollutant resulting from the olive oil industry, poses a serious ecological challenge due to its high pollution load. This effluent is highly concentrated in chemical oxygen demand (COD), which is 200 times higher than that of sewage wastewater. Moreover, OMWW is characterized by a strong acidity, high content of fatty matter, and high concentration of phenolic compounds.

In this study, coagulation/flocculation and filtration processes were investigated in order to decrease the pollution load of OMWW for potential reuse in olive orchard irrigation. First, two successive coagulation/flocculation steps were applied to a centrifuged OMWW. Lime and aluminum sulfate (alum) were used as coagulants by testing different concentrations in order to select optimal conditions. Then, the efficiency of various filtration systems using activated carbon and/or natural materials (olive stones, olive leaves, sand, Celite, and gravel) was tested. pH, electrical conductivity (EC), total solid (TS), and COD were measured before and after each treatment step (coagulation or filtration). Five phenolic compounds were monitored before and after applying the selected treatment steps under optimal conditions. The quantification and valorization of the sludge derived from coagulation/flocculation were also performed.

During the first coagulation/flocculation step, the results showed that the application of 8 g/L of lime combined with 7 g/L of alum allows the removal rates for EC, TS, and COD of approximately 10 %, 41 %, and 48 %, respectively. While the application of 5 g/L of lime and 4 g/L of alum during the second coagulation/flocculation step allows for lower reductions rates for TS (37 %) and higher reduction rates for COD (67 %). In addition, the resulting sludge showed its potential usage as a solid alternative fuel with a calorific value of 3295.79 cal/g. Moreover, filtration using activated carbon and gravel was found to be the optimum filtration system. The reduction rates were 51 %, 37 %, and 26 % for EC, TS, and COD, respectively.

Finally, the combination of coagulation/flocculation and filtration allows the substantial elimination of the studied phenolic compounds with global reduction yields of 97 % for vanillyl alcohol, 92 % for tyrosol, and 91 % for vanillic acid and p-coumaric acid.

Besides, Mediterranean countries are suffering from water shortages and the majority of olive mill trituration units are known for their artisanal types thus facing economic challenges. This research suggests a practical treatment process and the final effluent can be used to irrigate olive orchards at a rate of 170 m^3^ per hectare.

## Graphical abstract

**Figure:**
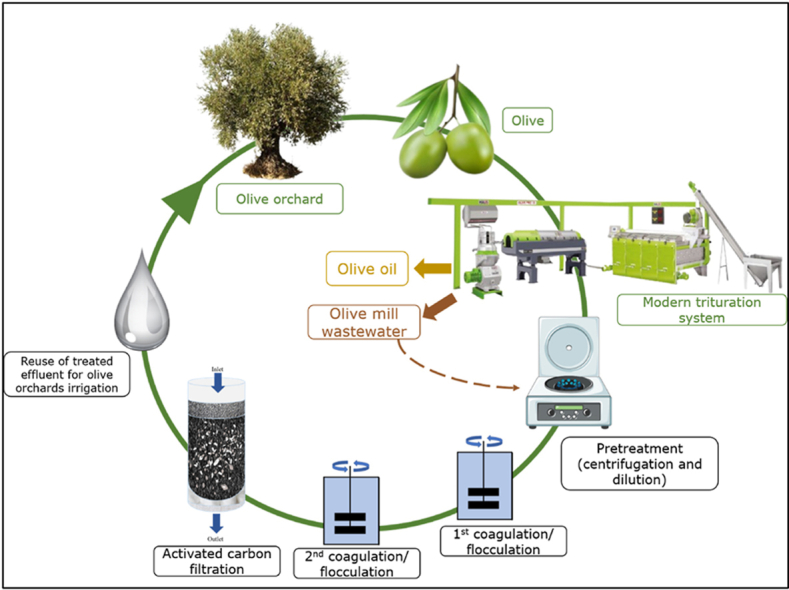

## List of abbreviations

AC: Activated carbon
Alum: Aluminum sulfate
COD: Chemical oxygen demand
EC: Electrical conductivity
EPA: Environmental Protection Agency
FOMWW: Filtered olive mill wastewater
HPLC: High-performance liquid chromatography
IR: Infrared
ISO: International Organization for Standardization
OL: Olive leaves
OMWW: Olive mill wastewater
OS: Olive stones
OWW: Olive washing wastewater
TOC: Total organic carbon
TS: Total solid
TSS: Total suspended solids

## 1 Introduction

Agroindustrial sectors are among the major water consumers [1]. A case study conducted in Cyprus showed that olive oil industries consume approximately 3.5 L of water to produce 1 L of olive oil [2]. In addition, a highly polluted effluent is generated. Olive mill wastewater (OMWW) is produced in a limited period and great volumes. This effluent contains approximately 200 g/L of chemical oxygen demand (COD) [3]. It has an acidic pH (4–5.8), a high phenol content (0.1–17.15 g/L) [4], and a high concentration of oil and grease (74 g/L) [5]. In addition, when OMWW is discharged into the environment, it could be an essential cause of eutrophication because it contains 60 mg/L of nitrogen (discharge legal limits: 1–2 mg/L) and 55 mg/L of phosphorous (discharge legal limits: 10–15 mg/L) [6,7]. Moreover, different heavy metals, such as iron, lead, copper, zinc, magnesium, cadmium, and nickel, were detected in OMWW, and they have a toxic effect on plant growth [4,[8], [9], [10]]. The origin and concentration of heavy metals are affected by different factors such as the location of the olive oil trituration unit (near industrial flue gases, iron steel factories, or areas known for their traffic pollution), cultivation and harvesting practices, and water quality used for irrigation or washing [[11], [12], [13], [14], [15]].

Thus, based on the composition of this effluent, direct discharge to natural resources is forbidden in different countries and it needs to undergo some preliminary treatments before reaching a wastewater treatment plant [16,17]. Different treatment methods for OMWW depollution have been studied and evaluated in the literature. The proposed treatments can be categorized into three.

• Physicochemical treatments such as coagulation/flocculation [3,18,19], filtration [[19], [20], [21]], liquid/liquid extraction [22,23], and adsorption [24,25].
• Chemical treatments, namely, electrocoagulation [26,27], Fenton, and ozonation [[28], [29], [30]].
• Biological treatments using aerobic or anaerobic digestion [20,31,32].

More specifically, different pretreatment methods were studied to improve the performance of the applied treatment. For example, the coagulation/flocculation method was applied to OMWW because it is a low-cost process [33,34]. In addition, different optimization techniques for coagulation/flocculation methods using aluminum sulfate (alum) and chitosan coagulants were tested [35]. Thus, the application of alum at 800 mg/L produced better performance than chitosan. Reduction rates of 62.89 %, 57.16 %, and 16.76 % were obtained for phenols, COD, and total organic carbon (TOC), respectively. Moreover, another study applied filtration and coagulation/flocculation as a pretreatment method for OMWW. Filters were composed of olive stones and led to reduction rates of 82.5 %, 73.8 %, and 11.3 % for total suspended solids (TSS), fatty matter, and total phenols, respectively. While removal rates of 31.3 % for COD and 10.8 % for total phenols were obtained after using 250 mg/L of ferric chloride as a coagulant and 5 mg/L of polyelectrolyte as a flocculant [19]. In a different aspect, filtration was documented as a pretreatment for OMWW depollution. Several filters were tested to decrease pollution load from OMWW [5]. Therefore, effluents were optimally treated by passing through three different filters of gravel, fine sand, and a mixture of acidified cotton and zeolite (weight ratio of cotton:clinoptilolite of 2:1), followed by treatment with activated carbon (AC) and lime. This treatment decreased turbidity, total fat, and electrical conductivity (EC) in OMWW by mean percentages of 96.8 %, 93.3 %, and 48.4 %, respectively. Thus, the use of integrated pretreatment processes can reduce costs and increase the efficiency of the final treatment step.

More recently, a seven-layer natural trickling filter was also tested in the literature. Coarse gravel, fine gravel, lime, sand, carbon char, sponge, and mesh were the materials used. Therefore, physical separation, filtration, coagulation, and adsorption were involved in this treatment. Reduction rates of 90 %, 69.8 %, 69 %, 41.6 %, and 40.2 % for phenol content, COD, TSS, EC, and biochemical oxygen demand, respectively, were shown [36].

Mediterranean countries suffer from OMWW because of their dense composition, great volumes produced in a limited period, and high-cost treatment. In addition, olive oil units are geographically widely dispersed [16], which makes the application of a centralized treatment challenging. Therefore, some countries tend to spread OMWW on agricultural soil for fertigation. To avoid unwanted side effects of OMWW spreading, it is crucial to follow the correct spreading techniques according to corresponding national legislations if available, spreading load, and time of spreading [37,38]. For instance, spreading should only be done on agricultural soil since soil tillage and agronomic practices accelerate the oxidative degradation of phenols.

Moreover, spreading should be avoided during the germination period of plants, away 10 m from rivers and water courses, and distributed uniformly on the soil. In addition, spreading load is a critical decision in wastewater application to soil, it is expressed as hectolitres per hectare. Spreading load could be calculated based on the polluting charge of the wastewater, which may be considered proportional to the total solid (TS) content following the “rule of 12” (Table 1) [38]. In addition, the spreading load can be increased by 50 % when applying on tree crops, such as olive groves and it can be substantially increased when a physicochemical pretreatment of OMWW occurs [38].

**Table 1:** The rule of 12 for OMWW spreading load [39].

Total solids (%)	3	4	5	6	7	8	9	10
Spreading load (hundreds of hectoliters of wastewater per hectar = tens of cubic meters)	9	8	7	6	5	4	3	2

The first line in Table 1 refers to TS concentrations in the OMWW, and the second line refers to the suggested quantity of OMWW that could be spread on the soil. Thus, the sum of two numbers from the same column is 12.

In the Lebanese context, olive millers tend to the direct disposal of olive liquid waste in the surrounding areas or in wells, rivers, lakes, digs, and valleys [40]. This behavior is due to the mismanagement and lack of wastewater treatment plants or even wastewater networks. Moreover, olive mill owners are known for their small family-type businesses with artisanal characteristics and they avoid treatment to prevent additional economic costs of olive oil production [40]. In addition, Lebanon has suffered from water shortage due to the longer dry season, overexploitation of fresh water, and challenges in sustaining the operation of water networks and wastewater treatment plants since the 2019 financial crisis [39].

Hence, on basis of the complex composition of OMWW, the infrastructure distribution of olive mills, the water shortages, and long dry season, a special attention was given to an integrated treatment system in the current research. Accordingly, coagulation/flocculation and filtration methods were studied in order to lower the pollution load of effluent. Our integrated treatment system aimed to.

i. Incorporate OWW in the pre-treatment step of raw-OMWW.
ii. Apply two steps of coagulation/flocculation on pre-treated OMWW using lime and alum as coagulants.
iii. Test different types of percolation filters for further elimination of pollutants.

In addition, physicochemical parameters, e.g. pH, TS, EC, PCs, and heavy metals were monitored.

## 2 Material and methods

### 2.1 Collection and preservation of OMWW

OMWW and OWW were collected from Said Saifan olive mills that are located in North Lebanon e.g. Koura Valley, which is 350 m above sea level and approximately 73 km from Beirut city. Samples were taken from a modern three-phase system and stored at 4 °C in plastic containers until analysis.

### 2.2 Chemical compounds and solutions

All chemicals used during this study were analytical grade and were used without further purification.

For the physicochemical analysis, potassium dichromate was purchased from Sigma-Aldrich. Calcium hydroxide (limewater) (95 %) and ammonium hydroxide were acquired from BDH Chemicals. Aluminum sulfate (90 %) was obtained from Lebanon Chemicals. Red methylene was from VWR International. Ferrous ammonium sulfate and ammonium chloride were purchased from Merck. Hydrochloric acid, sulfuric acid, and n-hexane were purchased from VWR/Fluka-Sigma-Aldrich, VWR, and Sigma-Aldrich, respectively. Ethanol and sodium hydroxide were purchased from Fischer Scientific.

For phenolic compound analysis, p-coumaric acid (98 %), vanillic acid (97 %), and ethyl acetate (≥99.7 %) were purchased from Sigma-Aldrich. Tyrosol (98 %) was obtained from Acros Organics. Vanillyl alcohol (98 %) was acquired from Alfa Aesar. Methanol (99.9 %) was obtained from Carlo Erba reagents, and formic acid (80 %) was purchased from Labosi.

Standard solutions used for HPLC analysis were prepared in acidified purified water (18.2 MΩ cm; Millipore) with HCl (pH 2–3). All other solutions were prepared using double-distilled water.

### 2.3 Analytical methods

#### 2.3.1 Physicochemical analyses

OMWW physicochemical analyses were conducted at the Industrial Research Institute (IRI) in Lebanon.

pH was measured using a pH meter “IQ 150” following ISO 4316. EC was measured using a Conductivity Meter based on ISO 7888. TS was determined following the total residue method described by EPA 160.3. COD was detected based on the potassium dichromate digestion method described by ISO 15705:2010.

Turbidity was measured using a portable meter Cyberscan IR: TB100 EUTECH instrument. Color analysis was performed using a spectrophotometer (UV 1601-Shimadzu) at 436 nm based on ISO 7887:1994. Ash was tested after incineration at 500 °C for 12 h of samples dried at 105 °C overnight. Minerals e.g. Pb, Fe, and Al were analyzed based on the atomic adsorption technique using an AA-7000 spectrophotometer.

#### 2.3.2 Phenolic compound analysis

Five phenolic compounds (PCs), namely, tyrosol, vanillyl alcohol, vanillic acid, p-coumaric acid, and gallic acid, were monitored. All analyses were performed at the University of Poitiers.

##### 2.3.2.1 Phenolic compound extraction method

PC extraction was performed before analysis using 5 mL of centrifuged (5000 rpm for 10 min), acidified (HCl, pH = 2–3) OMWW. The addition of 5 mL of n-hexane was performed for 10 min. After 2 h, the organic phase was used for PC extraction using 5 mL of ethyl acetate as a solvent and mixed for 10 min. Extraction was repeated thrice. The new organic phase containing the solvent and PC was subjected to distillation evaporation for solvent removal. Then, the dry residue was solubilized with 5 mL of a mixture composed of the initial mobile phase used for HPLC analysis (10 % methanol and 90 % purified water acidified with 0.1 % formic acid). Different dilutions with the initial mobile phase were taken at 1/10 for tyrosol analysis and ½ for vanillyl alcohol, vanillic acid, p-coumaric acid, and gallic acid analyses. Finally, under these conditions, 10 recovery tests of the five PCs were performed using different water matrices e.g. purified water, mineral water, river water, and wastewater. No significant change in the extract yield was observed regardless of the tested matrix. Similar results were then suspected in the OMWW. The extraction yields presented in Table 2 were considered during this study.

**Table 2:** Retention time, wavelength of detection, HPLC limits of quantification (LQ) and detection (LD) and extraction yields obtained for PC

Phenolic compounds	Retention time (min)	Wavelength of detection (nm)	LD-LQ in μmol/L *(mg/L)*	Extraction yield (%)
**Gallic acid**	3.4 ± 0.2	272	0.3–0.8 ***(0.05-0.14)***	89.7 ± 8.1
**Alcohol vanillyl**	5.6 ± 0.2	280	0.2–0.5 ***(0.03-0.08)***	85.8 ± 9.3
**Tyrosol**	6.3 ± 0.2	310	0.3–1.1 ***(0.04-0.15)***	82.9 ± 15.3
**Vanillic acid**	7.5 ± 0.3	292	0.2–0.6 ***(0.03-0.1)***	86.9 ± 12.0
**P-coumaric acid**	9.1 ± 0.3	227	0.3–1.0 ***(0.05-0.16)***	86.5 ± 11.1

Data are means of 4 ± SD. LQ: Limit of quantification; LD: Limit of detection.

##### 2.3.2.2 HPLC analysis of OMWW extracts

The analysis was performed on an Alliance HPLC system, equipped with a Waters 2998 diode array UV detector operating at 210 and 320 nm. The column used to analyze PC was an X-Bridge column (5 μm, 4.6 × 150 mm). Elution was performed at a flow rate of 1 mL/min using a mixture of water/formic acid (99.9:0.1 v/v) (A) and methanol/formic acid (99.9:0.1 v/v) (B) as a mobile phase.

For all samples, the injection volume was 100 μL and was analyzed using HPLC–UV either directly or after dilution in the mobile phase if necessary. The PC was quantified by external calibration using standards prepared in acidified purified water at 1, 2, 5, 10, 20, 50, 100, 200, and 500 μM. Triplicate measurements were conducted for each point in the range. Under these conditions, HPLC quantification limits of 0.5–1.1 μM were obtained according to the chemometric method proposed in the literature [41].

Retention time, specific wavelength of detection, and the HPLC quantification and detection limits obtained for each studied PC are shown in Table 2.

#### 2.3.3 Residue and sludge quantification

Residues derived from the centrifugation step and sludge derived from the coagulation/flocculation steps were quantified using the dry residue method:

*After centrifugation:*

Residues were separated for the dehydration step.

*After coagulation/flocculation steps:*

The decanted sludge was separated using a filtration pump and dehydrated on a hot plate. In both cases, further dehydration was performed in an oven at 105 °C for 12 h. The concentration of the dehydrated sludge was given in percent (w/v), as shown in equation (1) (Eq. (1))

(Eq-1) $$\text{Cs}\;\left(g/100\;\text{mL}\right)=\frac{Wf-We}{V*10}\text{,}$$

where.

Cs (g/100 mL): concentration of the dry residue/sludge per 100 mL of OMWW,

Wf (g): weight of total dehydrated residue/sludge and used beaker,

We (g): weight of the used beaker,

V (L): volume of the OMWW sample,

10: conversion factor.

#### 2.3.4 Residue and sludge valorization

After the quantification step, the valorization process was performed. Thus, the dehydrated residue/sludge was reused as an alternative fuel known as refuse-derived fuel. The gross calorific value of the dehydrated sludge represents the amount of heat released after sludge combustion, and it was measured using a calorimetric bomb (PARR 1266).

### 2.4 Experimental design for OMWW treatment

OMWW treatment experiments were conducted at IRI in Lebanon.

**Fig. 1:**
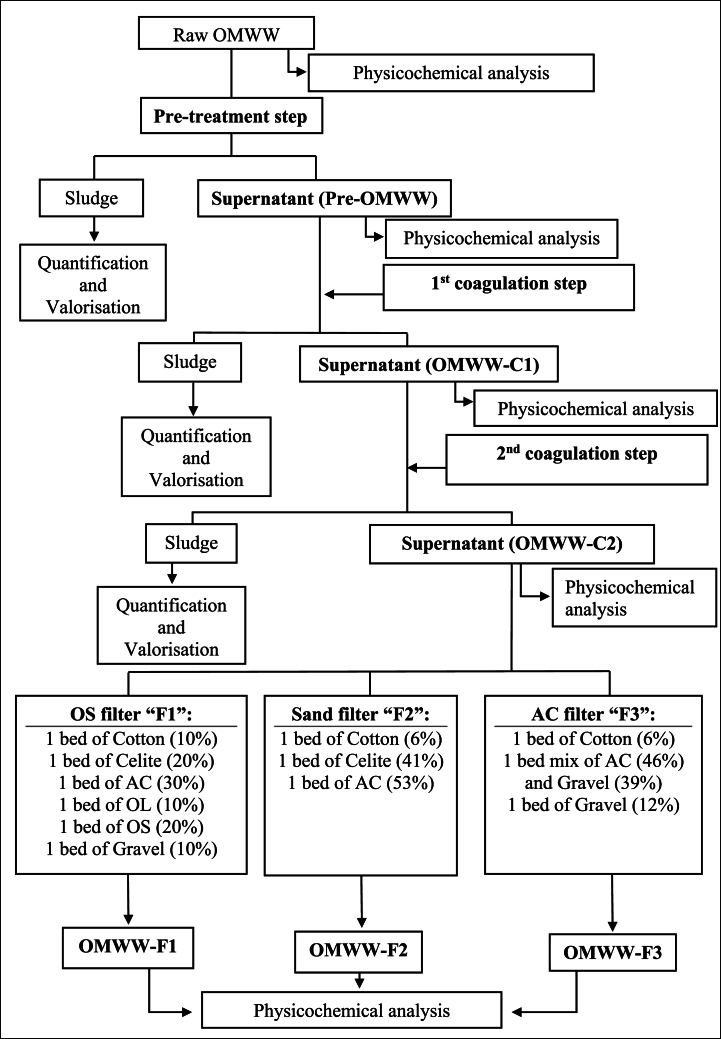
Experimental design followed for OMWW treatment resulted from modern trituration unit.

**Fig. 2:**
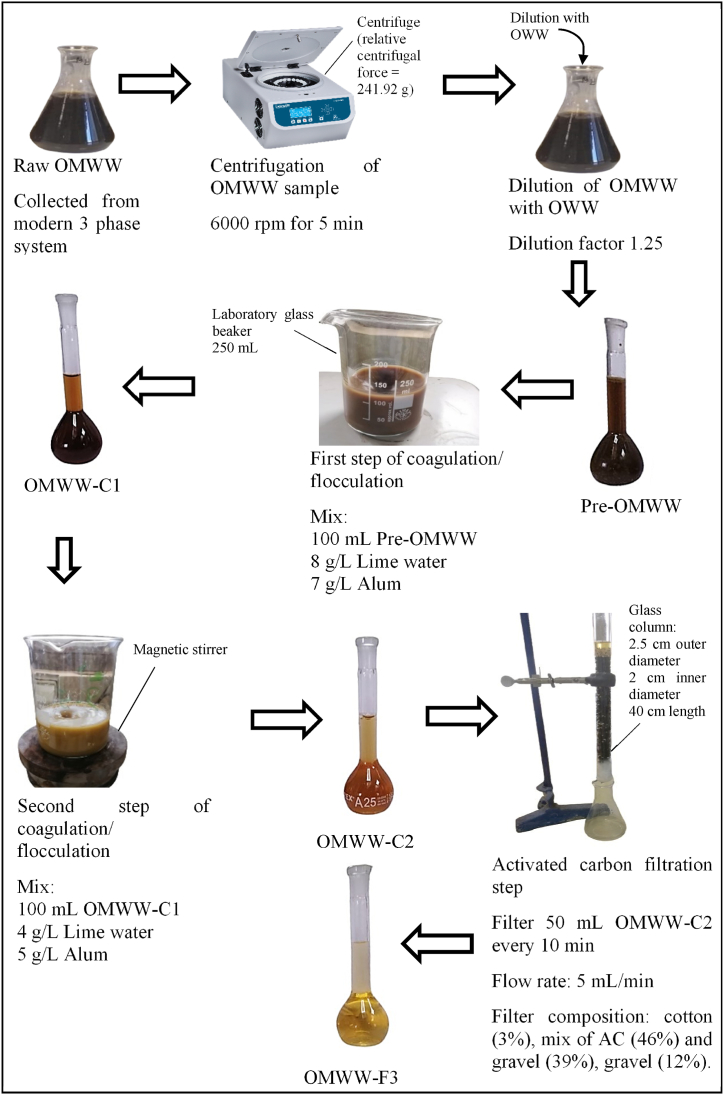
Photos of the real system used to treat OMWW at optimal conditions.

OMWW (100 mL) was used for the coagulation/flocculation experiments, and 50 mL of the resulting OMWW was used for filtration. Fig. 1 shows the experimental design of the successive applied treatment steps i.e., pretreatment, coagulation/flocculation, and filtration and Fig. 2 shows a real system used to treat OMWW. pH, EC, TS, and COD were measured during each treatment step to choose the optimal conditions. Additional parameters i.e. turbidity, color, ash, minerals “Pb, Fe, and Al,” and PCs were monitored under optimal treatment conditions. All parameters were determined to analyze the raw OMWW.

#### 2.4.1 Pretreatment step

To decrease the solid content of OMWW, centrifugation at 6000 rpm for 5 min i.e. relative centrifugal force = 241.92 g was performed. In addition, a dilution step was performed by adding 20 mL of OWW to 80 mL of OMWW. The resulting diluted supernatant corresponds to Pre-OMWW.

#### 2.4.2 Coagulation and flocculation

Two successive coagulation/flocculation steps were applied as shown in Fig. 1, Fig. 2. OMWW-C1 and OMWW-C2 were obtained from the first and second coagulation/flocculation steps, respectively.

Experiments were performed in 250 mL volume glass beakers. Samples (100 mL) were placed in beakers, and coagulants were added. Agitation was performed using an electric agitator with a magnetic stirrer. Continuously, rapid agitation was performed within 5 min to achieve a good dispersion of the reagents and a chemical destabilization of the colloids. Slow agitation was performed for 20 min to avoid the breaking of the formed flocs.

After 12 h of decantation, the supernatant was siphoned for physicochemical analysis and a second coagulation/flocculation treatment.

To determine the optimal conditions of the coagulation/flocculation step, two coagulants i.e. lime and alum were tested at different concentrations. During the first and second coagulation/flocculation steps, lime and alum (2 %) were tested at four different concentrations as shown in Table 3 and Table 4. One variable at a time was followed as an experimental design. Limewater was added first before the addition of alum.

**Table 3:** Physicochemical parameters of Raw-OMWW, OWW, Pre-OMWW, and OMWW-C1 resulted after the first step of coagulation/flocculation

Sample labelling	Coagulant/flocculant conc. (g/L)		Physicochemical characteristics						
	Limewater	Alum	pH	TS		EC		COD	
				Conc. (%) w/v	r (%)	Conc. (mS/cm)	r (%)	Conc. (g/L)	r (%)
**Raw-OMWW (n=2)**	**na**	**na**	4.83 ± 0.01	6.71 ± 0.83	**na**	8.91 ± 0.04	**na**	135.63 ± 2.55	**na**
**OWW (n=1)**	**na**	**na**	8.12	0.27	**na**	1.99	**na**	10.44	**na**
**Pre-OMWW (n=3)**	**na**	**na**	4.88 ± 0.08	3.1 ± 0.5	**na**	8.24 ± 0.33	**na**	84.79 ± 4.77	**na**
**OMWW-C1**									
S1 (n = 1)	1	3	10	2.92	**5**	8.42	**nd**	71.21	**16**
S2 (n = 1)	1	5	10.65	2.72	**12**	8.35	**nd**	63.76	**25**
S3 (n = 1)	1	7	9.48	2.55	**18**	7.83	**5**	74.50	**12**
S4 (n = 1)	1	9	9.12	2.26	**27**	7.7	**7**	74.22	**12**
S5 (n = 1)	2	3	11.54	2. 54	**18**	8.4	**nd**	57.49	**32**
S6 (n = 1)	2	5	12.12	2.58	**17**	8.39	**nd**	63	**26**
S7 (n = 1)	2	7	10.84	2.58	**17**	7.78	**6**	67.35	**21**
S8 (n = 1)	2	9	9.66	2.56	**17**	7.79	**5**	69.27	**18**
S9 (n = 1)	3	3	11.84	2.39	**23**	7.84	**5**	55.62	**34**
S10 (n = 2)	3	5	12.06 ± 0.12	2.27 ± 1.42	**27**	7.58 ± 0.46	**8**	54.6 ± 4.23	**36**
S11 (n = 2)	3	7	10.47 ± 0.08	2.27 ± 1.73	**27**	7.64 ± 0.28	**7**	51 ± 3.47	**40**
S12 (n = 1)	3	9	9.87	2.37	**24**	7.5	**9**	63.93	**25**
S13 (n = 2)	8	3	12 ± 0.07	2.37 ± 2.61	**24**	7.45 ± 0.58	**10**	52.28 ± 4.82	**38**
S14 (n = 2)	8	5	12.03 ± 0.15	2.21 ± 1.52	**29**	7.42 ± 0.41	**10**	49.23 ± 4.82	**42**
S15∗ (n = 2)	8∗	7∗	10.25 ± 0.11	1.82 ± 1.43	**41**	7.4 ± 0.38	**10**	43.79 ± 5.04	**48**
S16 (n = 2)	8	9	10.93 ± 0.07	1.87 ± 1.07	**40**	7.40 ± 0.63	**10**	52.69 ± 4.83	**35**

n, number of sample; Conc., concentration; r, reduction rate; na, not applicable; nd, not detected; ∗, optimal conditions.

**Table 4:** Physicochemical parameters and reduction rates of OMWW-C1 after the second step of coagulation/flocculation

Sample labelling	Coagulant/flocculant conc. (g/L)		Physicochemical characteristics							
	Lime water	Alum	pH	TS		EC		COD		
				Conc. (%) w/v	r (%)	Conc. (mS/cm)	r (%)	Conc. (g d’O_2_/L)	r (%)	
**OMWW-C1-S15 (n=2)**	**na**	**na**	10.25 ± 0.11	1.82 ± 1.43	**na**	7.4 ± 0.38	**na**	43.79 ± 5.04	**na**	
**OMWW-C2**										
T1 (n = 1)	1	2	11.27	1.57	**14**	5.25	**29**	29.51	**33**	
T2 (n = 1)	1	4	11.13	1.51	**17**	5.44	**26**	24.35	**44**	
T3 (n = 1)	1	6	10.11	1.52	**16**	5.76	**22**	29.25	**33**	
T4 (n = 1)	1	8	10.01	1.54	**15**	5.25	**29**	30.35	**31**	
T5 (n = 1)	3	2	12.82	1.49	**18**	5.24	**29**	20.98	**52**	
T6 (n = 1)	3	4	10.64	1.46	**20**	5.42	**27**	17.65	**60**	
T7 (n = 1)	3	6	11.83	1.47	**19**	5.92	**20**	24.83	**43**	
T8 (n = 1)	3	8	11.16	1.46	**20**	6.56	**11**	25.97	**41**	
T9 (n = 1)	5	2	12.72	1.43	**21**	7.13	**4**	15.11	**66**	
T10∗ (n = 2)	5∗	4∗	12.69 ± 0.16	1.15 ± 0.37	**37**	7.54 ± 0.43	**nd**	14.63 ± 2.72	**67**	
T11 (n = 2)	5	6	11.82 ± 0.05	1.17 ± 1.45	**36**	8.22 ± 0.35	**nd**	15.46 ± 5.35	**65**	
T12 (n = 2)	5	8	10.79 ± 0.17	1.25 ± 1.28	**31**	8.10 ± 0.46	**nd**	17.17 ± 4.62	**61**	
T13 (n = 1)	7	2	12.83	1.46	**20**	7.57	**nd**	16.07	**63**	
T14 (n = 2)	7	4	12.70 ± 0.18	1.48 ± 1.17	**19**	7.23 ± 0.46	**nd**	15.24 ± 4.35	**65**	
T15 (n = 2)	7	6	12.59 ± 0.08	1.49 ± 1.43	**18**	8.74 ± 0.53	**nd**	16.03 ± 5.03	**63**	
T16 (n = 1)	7	8	12.07	1.52	**16**	8.85	**nd**	17.47	**60**	

n, number pf sample; Conc., concentration; r, reduction rate; na, not applicable; nd, not detected; ∗, optimal conditions.

At optimal conditions, the coagulants’ concentration dosage was selected based on the maximum elimination of the COD.

#### 2.4.3 Filtration of OMWW using different filters

**Fig. 3:**
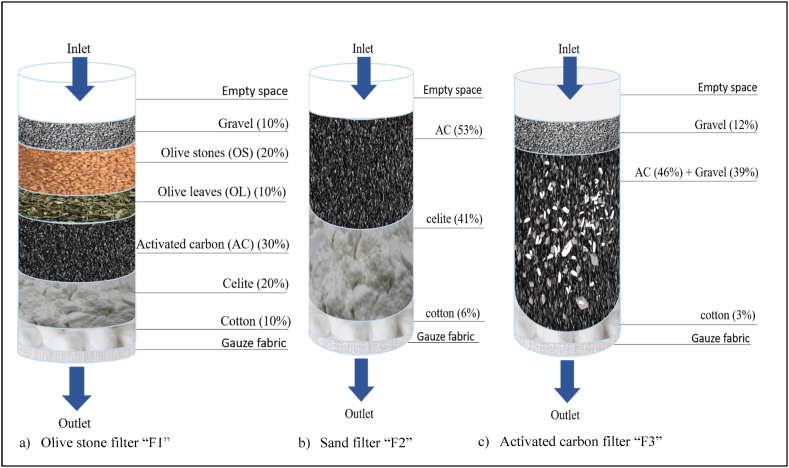
Schematic model of the filtration systems used for OMWW treatment.

After the first and second coagulation/flocculation, the supernatant i.e. OMWW-C2 was filtered through three different designed filtration systems (Fig. 3). Glass columns with an outer diameter of 2.5 cm, an inner diameter of 2 cm, and a length of 40 cm were used. Columns were sealed from the bottom with gauze fabric firmly held by tape. Materials filled the columns up to a height of 30 cm. The materials were composed of the following.

• OS filter “F1”: cotton (10 %), celite (20 %), AC (30 %), olive leaves (OL) (10 %), olive stones (OS) (20 %), and gravel (10 %) (Fig. 3a).
• Sand filter “F2”: cotton (6 %), celite (41 %), and AC (53 %) (Fig. 3b).
• AC filter “F3”: cotton (3 %), mixture of AC (46 %) and gravel (39 %), and gravel (12 %) (Fig. 3c).

The columns were placed vertically on a wooden support and kept at room temperature. A volume of 50 mL of OMWW-C2 was filtered every 10 min, and the effluent flow rate was recorded. Consequently, the flow rate of the AC filter “F3” was 5 mL/min, and lower flow rates were recorded for the olive stone filter “F1” (0.7 mL/min) and sand filter “F2” (0.5 mL/min).

## 3 Results and discussion

### 3.1 Physicochemical parameters of raw OMWW, Pre-OMWW, and OWW

The physicochemical characteristics of raw OMWW, Pre-OMWW, and OWW are presented in Table 3. Results show that all parameters of OMWW exceeded the legal limits for discharged wastewater outlined in the Lebanese decision nº8.1-2001 [42]. For raw OMWW, an acidic pH was noted at 4.83 ± 0.01. EC and TS were recorded at 8.91 ± 0.04 mS/cm and 6.71 ± 0.83 % (w/v), respectively. A high COD was noted at 135.63 ± 2.55 g d’O_2_/L. The pH of OWW was 8.12, and it had a COD of 10.44 g d’O_2_/L, 10 times lower than that of OMWW. Although OWW was less polluted than OMWW, its COD parameter also exceeded the legal limits for discharged wastewater outlined in the Lebanese decision nº8.1-2001 [42].

Pre-OMWW did not show a significant variation in pH, which remains at acidic conditions (pH = 4.88 ± 0.08) (Table 3). However, the pretreatment step leads to reduction rates of 54 % for TS, 8 % for EC, and 37 % for COD. Similarly, several studies conducted pretreatments before applying the coagulation process such as dilution, centrifugation, filtration, or acid cracking for gradual depollution of OMWW [43,44]. It was reported that better lime performance was observed for TS, phenols, reducing sugar, and nitrogen reduction when a centrifugation step was applied before the treatment of OMWW [44].

### 3.2 Coagulation/flocculation process

#### 3.2.1 First coagulation/flocculation step

Table 3 presents the effect of the first coagulation/flocculation step on the physicochemical characteristics of Pre-OMWW. Different concentrations of coagulant/flocculant were tested to select optimal conditions.

As shown in Table 3, Pre-OMWW, which was initially acidic (pH = 4.88 ± 0.08), became alkaline after the first coagulation/flocculation step, with pH varying between 9.12 and 12.12. It was also noted the pH increased with the lime concentration, and a reverse effect was noted when the concentration of alum increased. However, the pH was 10.25 when combining 8 g/L of limewater with 7 g/L of alum. In the literature, it was mentioned that alum is found predominantly in the form of Al(OH)_3_ when pH is approximately neutral, between 6 and 10, which enhances the adsorption phenomena [45].

TS removal increased when the concentrations of the coagulants i.e. limewater and alum increased. Thus, 41 % of TS elimination was noted when applying 8 g/L of lime and 7 g/L of alum. Moreover, in the literature, 63 % of TS was removed when lime was added to OMWW until its pH reached 12 [44]. It was discussed that lime can remove calcium ions, phosphorus, and suspended solids from wastewater. Lime can form calcium carbonate in an alkaline solution for suspended solid removal [46].

Moreover, EC removal decreased when the concentrations of alum increased. In addition, an average elimination of 10 % was recorded when applying 8 g/L of limewater with various alum concentrations (3, 5, 7, and 9 g/L). Similar results were obtained in the literature. However, at high concentrations of alum salts, lower EC values were observed [47].

In addition, COD removal increased when the concentrations of lime increased. COD elimination reached a maximum at an optimal alum concentration. Thus, 48 % was eliminated when combining 8 g/L lime and 7 g/L alum (S15). In the literature, the destabilization of colloidal forms when exceeding the optimum limit of coagulant concentration, was presented [48]. However, a COD reduction almost similar to our findings (45 %) was reported, when using lower concentrations of coagulants, namely, alum (5 g/L) and lime (3 g/L) [49]. This variation could be due to the different experimental conditions applied in the studies and the different organic charges of OMWW. In addition, coagulation/flocculation is highly dependent on the type and concentration of coagulants and wastewater composition [50]. Thus, it was reported that when wastewater contains more suspended particles, the zeta potential of the solution becomes unstable and requires higher doses of coagulant for treatment. The effect of coagulation/flocculation on OMWW treatment while monitoring different factors based on full-factorial design was studied [19]. Hence, they estimated which factor contributed more to the process. Thus, it was shown that the COD removal rate was essentially related to the coagulant type and coagulant/flocculant concentrations.

Thus, on the basis of these results, 8 g/L of lime and 7 g/L of alum were selected as optimal doses during the first coagulation/flocculation.

*Possible mechanism applied during the coagulation/flocculation treatment*.

**Fig. 4:**
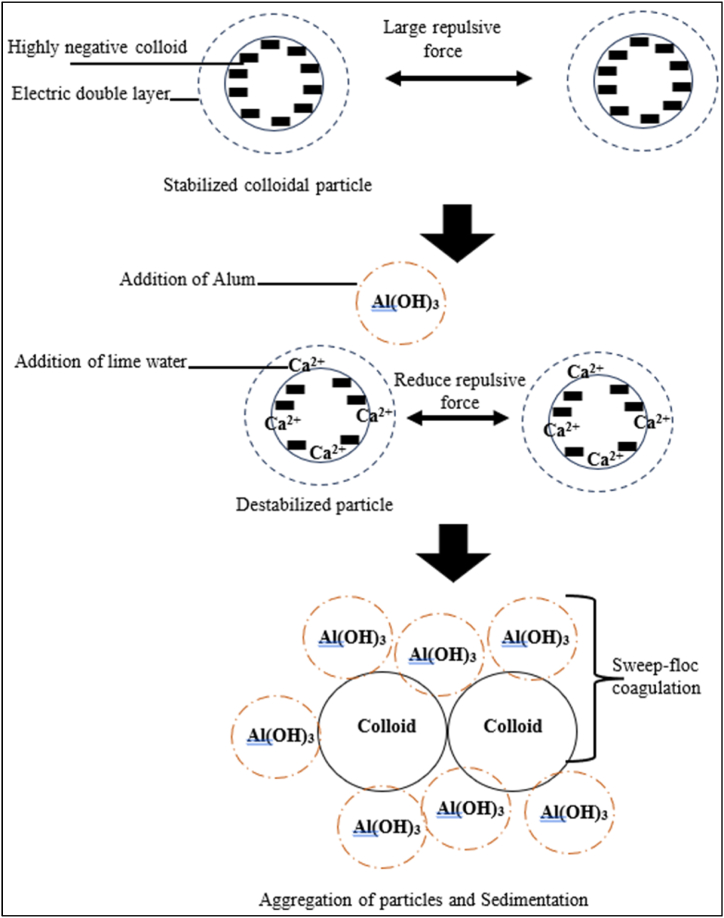
Illustrative figure of the coagulation/flocculation mechanisms using lime water and alum.

Fig. 4 shows the coagulation/flocculation mechanisms based on combining lime water and alum.

Limewater reacts with the natural alkalinity of water to form insoluble compounds when used as a coagulant in water treatment. These compounds precipitate out of the water, aiding in the removal of impurities. The process typically involves the following reaction (Eq. (2)):

(Eq. 2) $$Ca{\left(OH\right)}_{2}+CO_{2}\to CaCO_{3}+H_{2}O$$

where

Ca(OH)_2_: calcium hydroxide (lime)

CO_2_: carbon dioxide,

CaCO_3_: calcium carbonate (a precipitate).

As shown in Eq. (2), the formation of calcium carbonate helps to remove suspended and colloidal particles by binding them into larger flocs, which can then be removed by sedimentation or filtration [51]. The addition of lime can also increase the alkalinity of water, which results in a higher concentration of ions, some of which are positively charged. This mechanism is beneficial for coagulation processes as it can lead to the neutralization of negatively charged colloidal particles [52]. Therefore, the process of coagulation with lime involves the following steps.

1. Alkalinity increase: lime increases the water's alkalinity, providing the necessary conditions for coagulation.
2. Ion attraction: positively charged ions from lime interact with negatively charged colloidal particles. For instance, it has been reported that the Ca^2+^ ions can block the negatively charged groups of the surface molecules and act as bridges between functional groups of the two adjacent molecules. In addition, Ca^2+^ compresses the electrical double layer of the colloid particles [53,54].
3. Floc formation: the attraction between opposite charges leads to the aggregation of particles, forming larger clumps known as flocs.

Hence, in the current study, lime is used together with alum (aluminum sulfate) to enhance the coagulation process. In solution, alum is hydrolyzed to form aluminum hydroxide floc as well as hydrogen ions. Hydrogen ions will react with the alkalinity of water and decrease its pH (Eq. (3)), which explains the variation of pH in the current study when increasing the concentration of alum while fixing the concentration of lime (Table 3).

(Eq. 3) *Al*_*2*_*(SO*_*4*_*)*_*3*_*.18H*_*2*_*O* → *2Al*_*3*_ + *3SO*_*4*_^*−*^ + *18H*_*2*_*O→* 2Al*(OH)*_*3*_ + *6H*^*+*^ + *3SO*_*4*_^*2−*^ + *12H*_*2*_*O*−

Therefore, lime usage is required to ensure that the optimum coagulation pH is achieved. Consequently, colloidal matter is removed by adsorption onto/within the metal hydroxide that is formed and is sometimes referred to as sweep-floc coagulation [55]. Eq. (4) demonstrates the reaction between lime and alum coagulants when used at the same time [56]. This reaction can be applied during the coagulation/flocculation steps because both coagulants were used together to treat OMWW.

(Eq. 4) Al_2_(SO_4_)_3_ + 3(Ca)OH_2_ → 2Al(OH)_3_ + 3CaSO_4_

#### 3.2.2 Second coagulation/flocculation step

Table 4 shows the effect of the second coagulation/flocculation on the physicochemical characteristics of OMWW-C1.

The pH of the resulting OMWW-C2 varied between 10.01 and 12.83. Little pH increases were observed during the second coagulation/flocculation step, which reflects the saturation of the effluent with dissolved limewater coagulants.

Contrary to our observations during the first step of coagulation/flocculation, the elimination rate of TS was variable i.e. between 14 % and 37 % and did not follow a linear correlation depending on the concentration of alum and lime. A maximum reduction of TS was noted for sample T10 when applying 5 g/L of lime and 4 g/L of alum.

EC had the highest reduction rates (22%–29 %) when applying the minimum doses of limewater (1 g/L). No effect was seen for the highest doses (T9–T16). This approach is opposite to the case of the first step of coagulation/flocculation. This could be attributed to the fact that during the second coagulation/flocculation, the solution already contained some dissolved lime coagulant.

As shown in Table 4, the COD reduction rate varied between 31 % and 67 %. Similar observations were noted during the first step of coagulation/flocculation for the alum coagulant. For the lime coagulant, COD removal increased until reaching a limit (T13–T16). Thus, a maximum reduction rate of 67 % was noted when applying 5 g/L of limewater and 4 g/L of alum (T10).

Thus, on the basis of these results, optimal concentrations of limewater of 5 g/L and alum of 4 g/L were chosen during the second coagulation/flocculation step.

In addition, this step is crucial especially when the filtration step is applied immediately. Thus, the effluent received for filtration is less contaminated, which is crucial for preventing early filter blockage.

#### 3.2.3 Residue and sludge quantification

After the centrifugation step, 4.05 ± 0.11 % (w/v) of sludge was eliminated.

**Fig. 5:**
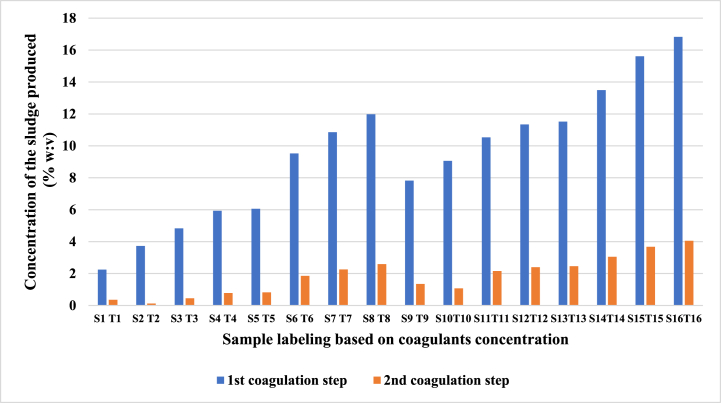
Concentrations of the sludge produced after the first and second coagulation/flocculation steps.

Fig. 5 shows the concentration of the produced sludge after the first and second coagulation/flocculation steps. It was observed that the concentration of the produced sludge was positively correlated with the coagulant concentration. However, lime usage had a stronger contribution to sludge production than alum. After the first coagulation step under optimal conditions (S15), 6.73 % (w/v) of sludge was obtained. However, lower sludge concentrations were generated (1.07 %, w/v) after the second step of coagulation under optimal conditions (T10).

Hence, the use of polymers can reduce the volume of sludge produced by making it more compact [57]. A reduction of 60%–70 % of the produced sludge was obtained (40 mL/L), after adding a cationic polymer of 5 mg/L of C-496 to 100 mg/L of alum [58].

However, the production of sludge as a secondary pollutant from the coagulation/flocculation process is considered one of the challenges faced by treatment facilities [59]. Sludge disposal can cost the treatment plant more than half of the treatment, up to 60 % of the total operating costs in wastewater treatment [59,60]. Therefore, the valorization of the sludge volume produced was conducted in this study (paragraph 3.3).

### 3.3 Residue and sludge valorization as carburants

After testing the sludge calorific value based on the calorimetric bomb technique, it was observed that the sludge after the centrifugation step had a calorific value of 7207.5 cal/g, and after the first coagulation/flocculation, it was 3295.79 cal/g. Controversially, sludge derived from the second coagulation/flocculation did not have a calorific value. Interestingly, a study compared different mixtures of alternative fuels composed of municipal solid waste, nonhazardous industrial waste, and agricultural waste. Consequently, the selected wastes had a high calorific value varying between 2601 and 8657 cal/g [61]. Moreover, the gross calorific values for the solid olive residue were within the range of 4953.75–6113.75 cal/g [62,63]. In addition, crude oil has a gross calorific value of 10531.0 cal/g [62].

### 3.4 Filtration processes

Table 5 shows the physicochemical characteristics of OMWW after passing through different types of filters. OS filter “F1” produces OMWW-F1. At the end of this filtration stage, the pH decreased from 12.69 to 6.13. This reduction is probably due to the release of organic acids in this filter. For instance, it has been shown that olive leaves are composed of 58.46 % polyunsaturated fatty acids, 46.7 % linolenic acid, and 30.21 ± 0.31 mg GAE/g of total PCs [64]. In addition, no reduction rate was observed for the EC, TS, and COD. This could be due to the organic contamination of the effluent with the filter's materials, especially because COD increased from 14.63 to 27.59 g d’O_2_/L. Similarly, in the literature, a filter using only OS as a material was designed [19]. Thus, it was seen that OS plays a major role in suspended solid and fat removal. However, a lower elimination of COD and total PCs has resulted. This could be attributed to the fact that only adsorbed molecules on suspended solids are removed.

**Table 5:** Physicochemical parameters and reduction rates of OMWW-C2 after filtration steps

	pH	TS		EC		COD	
		Conc. (%) w/v	r (%)	Conc. (mS/cm)	r (%)	Conc. (g/L)	r (%)
**OMWW-C2-T10 (n=2)**	12.69 ± 0.16	1.15 ± 0.37	**na**	7.54 ± 0.43	**na**	14.63 ± 2.72	**na**
**OMWW-F1 (n=1)**	6.13	2.72	**nd**	10.89	**nd**	27.59	**nd**
**OMWW-F2 (n=1)**	11.12	1.11	**3**	6.49	**14**	14.03	**4**
**OMWW-F3 (n=5)**	8.82 ± 0.32	0.72 ± 0.09	**37**	3.69 ± 0.51	**51**	10.76 ± 0.15	**26**

Conc., concentration; r, reduction rate; na, not applicable; nd, not detected.

Sand filter “F2” gives OMWW-F2 with a lower pH (11.12) compared with OMWW-C2 (12.69). This reduction could be affected by the pH of Celite, which is approximately 10. Moreover, the evolution of EC during this filtration step showed a reduction from 7.54 to 6.49 mS/cm (Table 5). This decrease could be attributed to the fact that minerals were fixed on the sand particles. Similarly, it was discussed that adsorption and ion exchange are the principal physicochemical surface reactions that occur on sand during the transit of wastewater through this medium [65]. In addition, the analysis of TS and COD showed small reduction rates of 3 % and 4 %, respectively. In the literature, it was reported that the elimination of organic matter is possible based on physical phenomena e.g. sedimentation and filtration of the particulate forms and biological degradation of the dissolved particulate using bacterial flora [65]. Accordingly, biological degradation is essentially applied in the upper soil layers where oxygenated conditions are generally dominant [66]. However, it was shown a better reduction rate of COD (79.75 %) after applying a sand filter for 11 months [65].

For the AC filter “F3,” a pH reduction was seen from 12.69 to 8.82 in OMWW-F3 (Table 5). It was discussed that AC can influence the pH of a solution by adsorbing and trapping certain compounds, such as acids and bases, due to the porous surface (up to 1500 m^2^/g) [67,68]. In this study, lime trapping could explain the decrease in the pH solution. In addition, carbon showed its ability to release carbonic acids when it is dissolved in water and reacted with hydrogen ions [68]. Moreover, EC, TS, and COD showed reduction rates of 51 %, 37 %, and 26 %, respectively. These decreases were due to the ability of AC to reduce colloidal and soluble organic particles, especially because natural organic matter with a molecular weight of less than 5 KDa is readily adsorbed by granular activated carbon [5,69,70].

*Possible mechanisms applied during the filtration processes*

**Fig. 6:**
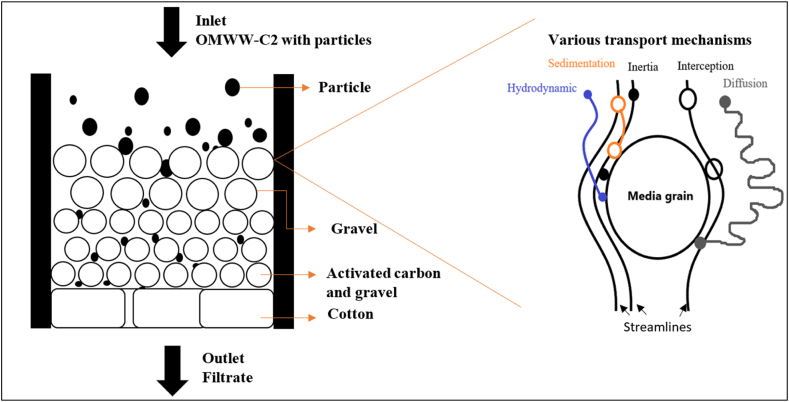
Illustrative figure of the deep filtration process and transportation mechanisms applied during multi-media filtration.

Fig. 6 shows the deep filtration process and the transportation mechanisms applied during the multi-media filtration.

The removal of particles by media filtration occurs by deep-bed filtration. It was discussed that during deep-bed filtration, two stages dominate when wastewater passes through the filter medium [71]. In the initial stage, particles are attached to the clean filter media. Following this stage, a transient stage occurs, where particles are deposited on the surface media partially covered by already deposited particles. This stage encompasses the ripening and working stages in which particles are removed at a constant rate. After this stage, the removal of particles decreases and we reach the “breakthrough stage.”

In addition, during filtration and particle removal, two main mechanisms are highlighted, namely, particle transport and particle attachment [72,73].

First, particles in suspension are transported near filter grains i.e. media based on different mechanisms: sedimentation, interception, diffusion, inertia, and hydrodynamic effect [71]. These transport mechanisms have a crucial role in filtering all particles, especially the ones that are smaller than the filter pores. It was seen that because of the transport mechanisms, particles move across the water streamlines and arrive adjacent to a media grain surface.

Second, the effective removal of these particles is related to the attachment mechanism, which depends on the surface forces acting between particles and filter grains when their separation distance is in the order of nanometers [71]. Thus, when the separation distance is up to 100 nm, long-term forces are dominant and are subdivided into two forces: van der Waals and electric double-layer forces e.g. repulsive or attractive. In addition, when the separation distance is up to 5 nm, short-term forces are dominant and are subdivided into two forces as well: the Born and Hydration forces. The importance of chemical alterations that influence the attachment of solids in the bed filter, was highlighted in the literature [74].

Interestingly, a particular force “the mutual adsorption force” was discussed when using coagulation/flocculation treatment before filtration [75]. More precisely, particle attachment to a filter grain or to the particles associated with the filter grain can arise from the mutual adsorption of dissolved coagulants, such as the hydrolysis products of alum. These products form links, having one end attached to the filter grain surface and the other to the particle. In addition, it was reported that particles and media are naturally negatively charged and repelled by each other. Therefore, in the current study, the coagulation process was crucial to be applied before filtration to change the surface chemistry of target particles and enhance filtration.

Moreover, all of the abovementioned surface forces e.g. van der Waals force, electric double-layer force, Born force, and hydration force will form a resultant adhesive force on the particle because of their interaction with the filter media [71]. Thus, this force is attractive between particles and media grains when the distance between them is close and repulsive when the distance increases. However, when the separation distance is increased further, the adhesive force can either reach zero or become attractive again. Therefore, these forces could be presented during the filtration of OMWW-C2.

In addition, filtration mechanisms are affected by the type of media. For instance, the best removal effect was seen when using filter “F3.” It was discussed that AC is considered the best candidate to adsorb different organic (phenol, glucose, and sucrose) and inorganic (heavy metals) water contaminants [76].

### 3.5 OMWW physicochemical characteristic after integrated treatment processes under optimal conditions

#### 3.5.1 Turbidity, color, ash, and mineral reduction after integrated treatments

Table 6 shows the physicochemical parameters of OMWW as well as the reduction rate after the integrated treatments applied at optimal conditions.

**Table 6:** Physicochemical parameters and reduction rates of OMWW after integrated treatment processes at optimal conditions

	Regulation	Raw-OMWW (n = 2)	Pre-OMWW (centrifugation + dilution) (n = 3)		OMWW-C1 (First coagulation) (n = 2)		OMWW-C2 (Second coagulation) (n = 2)		OMWW-F3 (AC filtration) (n-5)		GRR (%)
		Conc.	Conc.	r (%)	Conc.	r (%)	Conc.	r (%)	Conc.	r (%)	
**Turbidity (NTU)**	ND	27.40 ± 1.53	12.18 ± 3.25	**56**	1.36 ± 3.85	**89**	0.36 ± 0.25	**74**	0.28 ± 0.04	**22**	**99**
**Color (at 436 nm)**	ND	390.7 ± 0.14	324.1 ± 0.08	**17**	146.9 ± 0.32	**55**	40.5 ± 0.12	**72**	20.06 ± 2.65	**50**	**95**
**Ash (%;m/v)**	ND	0.91 ± 0.03	0.74 ± 0.02	**19**	0.85 ± 0.24	**nd**	0.59 ± 0.08	**31**	0.25 ± 0.07	**58**	**73**
**Pb (μg/L)**	1000^1^	61.52 ± 0.06	31.11 ± 0.06	**49**	10.22 ± 0.10	**67**	8.68 ± 0.06	**15**	4.32 ± 1.30	**50**	**93**
**Fe (μg/L)**	5000^1^	7.74 ± 0.41	5.73 ± 0.35	**26**	2.76 ± 0.27	**52**	1.41 ± 0.28	**49**	2.45 ± 0.86	**nd**	**68**
**Al (μg/L)**	10^1^	160 ± 0.34	140 ± 0.31	**13**	160 ± 0.25	**nd**	140 ± 0.18	**13**	120 ± 0.01	**14**	**25**

Conc., concentration; r, reduction rate; nd, not detected; ND, not determined; GRR, Global Reduction Rate; 1, Limits of the physicochemical characteristics of wastewater discharge in a treatment plant, based on the Lebanese decision 8.1–2001.

##### 3.5.1.1 Pretreatment step

During the pretreatment step, more than half of the turbidity was reduced (56 %). In addition, reduction rates of 17 % and 19 % were noted for color and ash content, respectively. Furthermore, reductions of 49 %, 26 %, and 13 % were obtained for Pb, Fe, and Al, respectively. It was discussed that there is a linear relationship between the sedimentation rate and the density of particles to be separated during centrifugation [77]. For instance, the densities of Pb, Fe, and Al are 11.34, 7.874, and 2.7 g/cm3, respectively [78].

##### 3.5.1.2 First step of coagulation/flocculation

After the application of the first coagulation/flocculation, a significant reduction in turbidity (89 %) was noted. It was discussed that coagulation/flocculation treatment reduces the turbidity of the solution after colloidal destabilization and floc formation, which will settle [50]. In addition, a color reduction of 55 % was observed. However, no reduction in ash content was detected. Likewise, the composition of minerals shows a reduction of 67 % for Pb and 52 % for Fe with a release of 20 μg/L for Al.

##### 3.5.1.3 Second step of coagulation/flocculation

After the second coagulation/flocculation, a turbidity reduction of 74 % was recorded. Similarly, a color reduction of 72 % was noted, from brown to light yellow. In the literature, color change has been attributed to limewater, which was effective in lightning the color of OMWW [5]. Hence, ash had a reduction of 31 %. However, reductions of 15 %, 49 %, and 13 % were obtained for Pb, Fe, and Al, respectively.

### 3.6 Filtration step using an AC filter (F3)

During this filtration step, reductions of 22 %, 50 %, and 58 % were obtained for turbidity, color, and ash content, respectively. It was shown that AC has a bleaching effect on solutions [5]. Concerning the minerals, reductions of 50 % and 14 % were noted for Pb and Al, respectively. No elimination was observed for Fe.

#### 3.6.1 Reduction of PCs after integrated treatments

Table 7 shows the variations in the five studied PCs present in OMWW before and after specific treatments.

**Table 7:** Phenolic compounds concentrations in different types of treated OMWW at optimal conditions after integrated treatments

	LD-LQ in μmol/L (mg/L)	*Raw-OMWW*	*Pre-OMWW*		*OMWW-C1*		*OMWW-C2*		*OMWW-F3*		GRR (%)
		*Conc. (n=3)*	*Conc. (n=3)*	*r (%)*	*Conc. (n=3)*	*r (%)*	*Conc. (n=3)*	*r (%)*	*Conc. (n=3)*	r (%)	
**Gallic acid d (1/2)**	2.6–8.6 (0.4–1.5)	*< LD*	*<LD*	***nd***	*<LD*	***nd***	*<LD*	***nd***	*<LD*	**nd**	**nd**
**Vanillyl alcohol d (1/2)**	0.4–1.1 *(0.1 – 0.2)*	357 ± 40 *(55 ± 6)*	214 ± 24 *(33 ± 4)*	**40**	49 ± 6 *(8 ± 1)*	**77**	12 ± 2 *(2 ± 1)*	**76**	10 ± 2 *(2 ± 1)*	**17**	**97**
**Tyrosol d (1/10)**	3.8–12.5 *(1 – 2)*	2067 ± 391 *(286 ± 54)*	2364 ± 445 *(327 ± 61)*	**nd**	1051 ± 204 *(145 ± 28)*	**56**	543 ± 110 *(75 ± 15)*	**48**	156 ± 39 *(22 ± 5)*	**71**	**92**
**Vanillic acid d (1/2)**	0.5–1.4 *(0*.*1 – 0*.*2)*	124 ± 20 *(20 ± 3)*	159 ± 25 (27 ± 4)	**nd**	39 ± 7 *(7* ± 1*)*	**75**	24 ± 5 *(4)*	**38**	11 ± 3 *(2* ± 1*)*	**54**	**91**
**P-coumaric acid d (1/2)**	0.7–2.2 *(0*.*1 – 0*.*4)*	111 ± 16 *(18 ± 3)*	142 ± 20 *(23 ± 3)*	**nd**	21 ± 4 *(3 ± 1)*	**85**	9 ± 3 *(1 ± 0*.*5)*	**57**	10 ± 3 *(2 ± 0*.*5)*	**nd**	**91**

These values take into consideration the extraction yield and the dilution used for the HPLC analysis (i.e. HPLC limits x dilution factor/extraction yield). nd, not detected; LD, Limit of detection; LQ, Limit of quantification; d, dilution used during the analysis; GRR, Global Reduction Rate.

Gallic acid was below the detection limit (0.05 mg/L) in raw OMWW.

##### 3.6.1.1 Pretreatment step

During the pretreatment stages, only 40 % of vanillic alcohol was eliminated, with no reduction yield noted for the other PC.

##### 3.6.1.2 First step of coagulation/flocculation

After the first coagulation/flocculation, significant reductions in vanillic alcohol (77 %), vanillic acid (75 %), and para-coumaric acid (85 %) were obtained. In addition, more than half of the presented tyrosol was reduced (56 %). In the literature, a reduction varying between 10 % and 19 % in total phenols was shown after the application of coagulation/flocculation [19,79,80]. Reduction rates were influenced by the initial concentrations of phenols and PCs and the experimental conditions of the applied coagulation/flocculation process.

##### 3.6.1.3 Second step of coagulation/flocculation

After the second coagulation/flocculation, reductions of 76 %, 48 %, 38 %, and 57 % were recorded for vanillic alcohol, tyrosol, vanillic acid, and para-coumaric acid, respectively.

##### 3.6.1.4 Filtration step using an AC filter (F3)

During the AC filtration step, a significant reduction of 71 % of tyrosol was obtained. Furthermore, reductions of 54 % and 17 % were recorded for vanillic acid and vanillic alcohol, respectively. No further elimination of para-coumaric acid was observed. It has been discussed that the pore size of granular AC (especially at 0.89 nm) can filter phenols through adsorption [81]. Furthermore, it has been reported that the adsorption of PC on AC is influenced by several factors [82].

• Speciation of the molecules: If the pH > pKa, the compounds and the surface of carbon are mainly negatively charged. Therefore, electrostatic repulsion leads to a reduction in the adsorption capacity. Thus, a better elimination of the PCs is performed at a lower interval because at pH < pKa, the PCs are mainly in neutral molecular form.
• Solubility of molecules: The higher the solubility is, the weaker the adsorption.
• Size of the molecules: better adsorption of small PC on AC.
• Nature of functional groups: it has been mentioned that PCs with acid functions have a better ability to be attracted by AC because they have a carboxy group on the aromatic ring, which lowers its electron density and thus facilitates the action of the aromatic nucleus as an acceptor.

In the case of tyrosol, with high pKa values and one of the highest log P values of the PC studied, these results are therefore in agreement with the data in the bibliography. However, tyrosol is the most concentrated PC in OMWW and the adsorption phenomena depend on the respective concentrations of the substrates and the phenomena of competition with the natural organic matter.

## 4 Strengths, weaknesses, opportunities, and threats (SWOT) analysis of the studied OMWW integrated treatments

SWOT analysis is a business strategy tool that identifies and evaluates the strengths, weaknesses, opportunities, and threats of a project. Thus, it includes the internal and external factors influencing the successful implementation of a treatment technology, which will affect the decision taken by the owners for the management of their business. In the SWOT method, internal factors include strengths and weaknesses and external factors include the opportunities and threats of a project or business. In addition, the threats, opportunities, weaknesses, and strengths i.e. TOWS analysis, which has the same acronyms as SWOT, identifies interactions between factors when connecting them. As a result of its application, it is possible to show whether strengths can avoid external threats and capitalize on opportunities and whether weaknesses can be eliminated by taking advantage of external opportunities and how to avoid both weaknesses and threats [83].

In this study, a SWOT analysis was applied for a full overview of the performance of the tested treatment and to evaluate it from different perspectives. In addition, SWOT analysis was supplemented with TOWS analysis for a better vision of the treatment performance and to define further recommendations and strategies.

### 4.1 Internal factors (strengths and weaknesses)

Internal factors are presented in Table 8. It must be said that coagulation/flocculation and filtration on AC are practical treatments suitable for a rapid installation and maintenance. The applied integrated treatment was considered an eco-friendly system based on the use of safe materials e.g. AC and limewater. However, the use of alum solution was controlled and no detection of aluminum release in the effluent was seen after the second coagulation/flocculation system. In addition, the coagulation/flocculation process was considered a circular economic process, especially because sludge treatment was tested for its calorific value. In addition, the studied treatments allowed OMWW to be less toxic to the environment when discharged due to a crucial decrease in COD (92 %). This act will conserve the environment and protect watersheds.

**Table 8:** SWOT (strengths, weaknesses, opportunities, threats) for OMWW proposed treatment.

Strengths	Weaknesses
S1: Practical treatment S2: Eco-friendly system S3: Circular economic system S4: Added value to the society: protecting the ecosystem, preventing river pollution.	W1: OMWW quality fluctuation W2: Uniqueness of the technology W3: Lack of additional analysis for implementation at a larger scale

**Opportunities**	**Threats**

O1: AC regeneration O2: Use of the water for olive orchards irrigation and fertigation O3: Benefit from the PC O4: Increasing interest in green technology	T1: Weak enforcement of the current legislation (lack of a strict regulation) weak regulations and law interventions T2: OMWW availability T3: Income instability T4: Weak financial position of the majority of olive mill owners

In addition, system weaknesses were attributed to the fact that OMWW has fluctuating physicochemical characteristics. Thus, pH monitoring before coagulation/flocculation should be controlled. Moreover, the implemented technologies are well known before. Treatment applications at a pilot scale were missed from the current study.

### 4.2 External factors (opportunities and threats)

Because the world is moving toward green technology and by-product valorization, there is a great opportunity to exploit treatment by-products. Thus, AC media can be recycled and reused. Treated OMWW can be spread on olive groves. According to the “rule of 12,” as explained by Peri C. and Proietti P. [39], the spreading load of the resulting effluent from the current study would be equal to 170 m^3^ per hectare. More precisely, the final TS concentration was equal to 0.72 % which corresponds to a spreading load equal to 113 m^3^ per hectare, in addition to 50 % since it is destined for crop irrigation [39]. To put it in perspective, a super high-density olive grove needs a minimum of 2500 m^3^ of water per hectare [84]. Moreover, additional studies can be performed for the desorption processes of the PC presented in sludge after coagulation/flocculation or filtered media.

The major threat is the absence of strict legal enforcement and penalties for environmental infringements in combination with the large volume of OMWW at the end of the year. In addition, the fluctuation of olive oil production every season leads to income instability in addition to the financial difficulties of the majority of the olive mill owners.

### 4.3 TOWS analysis

Table 9 shows the TOWS analysis that indicates conceptual strategies and interactions between factors.

**Table 9:** TOWS (threats, opportunities, Weaknesses, Strengths) analysis of OMWW treatment

	Strengths	Weaknesses
**Opportunities**	**SO** (1) Re-invest profits in AC regeneration (O1, S1) (2) Increase investment in marketing for the green treatment applied to expand the market (O2, O3, O4, S2, S3) (3) Increase treatment efficiency by increasing its shelf-life and regenerating its materials (O1, S4)	**WO:** (1) Enter new markets for treatment application at larger scale (O4, W2, W3) (2) Take advantage of the by-products (O1, O3, W2)
**Threats**	**ST:** (1) Offer payment facilities for treatment implementation (T3, T4, S1, S3) (2) Storage of the available OMWW samples for future treatment (T2, S4) (3) Apply pressure on the state for treatment application and nature conservation (T1, S4)	**WT:** (1) Highlight OMWW pollution effect and toxicity on the eco-system (T1, T2, W1) *Overcome weaknesses by making them strengths*

SO, Strengths/Opportunities; WO, Weaknesses/Opportunities; ST, Strengths/Threats; WT.

Thus, a maxi–maxi strategy represents the interaction between SO i.e. strengths/opportunities. The main focus of increasing circular-treatment application with the merging interest in green technology was related. A mini–maxi strategy highlights the relation between weaknesses and opportunities. Therefore, taking advantage of the by-products and new markets is essential for overcoming treatment weaknesses. A maxi–mini strategy is applied in the strengths and threats category. Thus, offering payment facilities can overcome the threat to olive millers’ financial status. A special emphasis was given to environmental conservation by exerting pressure on the state to implement clear regulations and applications. Finally, a mini–mini strategy focuses on overcoming the weaknesses and threats to have a successful treatment application. Thus, the main focus on OMWW toxicity is essential to encourage treatment application.

## 5 Conclusion

The results of this study indicated that coagulation/flocculation and filtration on AC filter should be considered as a potent integrated treatment system for OMWW. It requires two coagulation steps and one step of filtration to achieve 92 %, 99 %, 95 %, 93 %, and 89 % of COD, turbidity, color, Pb, and TS removal, respectively. This treatment system affected as well PCs with 97 %, 92 %, 97 %, and 91 % of abatement rate for vanillic acid, tyrosol, vanillyl alcohol, and p-coumaric acid, respectively.

A complete characterization of water before and after treatment could be considered for future research, such as, transient parameters e.g. alkalinity, acidity, hardness, sulfates, chlorides, phosphates, nitrates, and calcium. In addition, AC media could be analyzed in order to study the adsorption isotherms. In addition, a future study could be performed to evaluate the effect of the treated effluent on olive orchard irrigation.

## CRediT authorship contribution statement

**Layla Moustafa Fleyfel:** Writing – original draft, Visualization, Software, Resources, Project administration, Methodology, Investigation, Formal analysis, Data curation. **Joseph Matta:** Visualization, Validation, Supervision, Resources, Conceptualization. **Nicole Fakhoury Sayegh:** Visualization. **Nasma Hamdi El Najjar:** Writing – review & editing, Visualization, Validation, Supervision, Project administration, Investigation, Funding acquisition, Formal analysis, Data curation, Conceptualization.

## Data availability statement

Data included in article/supp. material/referenced in article.

## Declaration of competing interest

The authors declare the following financial interests/personal relationships which may be considered as potential competing interests:Layla Fleyfel reports financial support was provided by Hubert Curien CEDRE Partnership 27 (PHC CEDRE),. Layla Fleyfel reports financial support was provided by 10.13039/501100005993National Council for Scientific Research of the Lebanese 28 Republic (10.13039/501100007175CNRS-L). If there are other authors, they declare that they have no known competing financial interests or personal relationships that could have appeared to influence the work reported in this paper.
